# Non-intact Families and Children’s Educational Outcomes: Comparing Native and Migrant Pupils

**DOI:** 10.1007/s10680-022-09638-z

**Published:** 2022-09-05

**Authors:** Raffaele Guetto, Francesca Zanasi, Maria Carella

**Affiliations:** 1 Department of Statistics, Computer Science, Applications, University of Florence, Florence, Italy; 2 Department of Political and Social Sciences, European University Institute, Florence, Italy; 3 Department of Political Sciences, University of Bari Aldo Moro, Bari, Italy

**Keywords:** Separation, Divorce, Single-parent households, Migration background, Education, Italy

## Abstract

This study explores whether the association between living in a single-parent household and children’s educational outcomes differs by migration background through comparing natives with first- and second-generation migrant pupils from different areas of origin. While there is strong evidence of an educational gap between migrant and native pupils in Western countries—and particularly in Italy—the interaction with family structure has been under-investigated. We suggest that native children have more socioeconomic resources to lose as a consequence of parental breakups, and thus may experience more negative consequences from living in a single-parent household compared to migrant children, who tend to have poorer educational outcomes regardless of family disruptions. Moreover, for migrant children, family disruption could result from parents’ migratory project (transnationalism) rather than separation or divorce, thus not necessarily implying parental conflict and a deteriorating family environment. Empirical analyses of data from the ISTAT ‘Integration of the Second Generation’ survey (2015) show that native Italian pupils from single-parent households in lower secondary schools are more strongly penalised in terms of grades, and less likely to aspire to the most prestigious upper secondary tracks when compared to second- and, especially, first-generation children. Indeed, the latter have been found to experience virtually no negative consequences from parental absence. Contrary to expectations, we found no substantial differences in the non-intact penalty based on the reason for parental absence (transnationalism vs divorce), nor by migrants’ area of origin.

**Supplementary Information:**

The online version contains supplementary material available at 10.1007/s10680-022-09638-z.

## Introduction

A bulk of empirical research has shown that growing up in a single-parent household is associated with adverse child outcomes in several domains, such as psychological well-being and health (Härkönen et al., 2017). These negative consequences also involve short-term educational performances (Amato & Anthony, 2014; Radl et al., 2017) and long-term educational attainment, such as the probability of obtaining a tertiary degree (Bernardi & Radl, 2014; Guetto et al., 2022), which can lead to an accumulation of disadvantages over the life course.

More recently, demographers and sociologists have focused their attention on the possible heterogeneity across social groups in the negative consequences of living in a non-intact family (‘non-intact penalty’). Parental socioeconomic status (SES) and ethnicity have often been identified as potential moderators of the association between parental absence and children’s educational outcomes (Aquino et al., 2022). Empirical evidence in this regard has been more mixed, especially as far as heterogeneity by SES is concerned. Some studies have found that socioeconomically advantaged families manage to shelter their offspring from the negative consequences of parental separation (Amato & Anthony, 2014; Grätz, 2015), whereas others have reported that children of high-SES parents tend to be more negatively affected (Nilsen et al., 2020). Bernardi and Boertien (2016) argued that children of advantaged families may have ‘more to lose’ in terms of social, cultural, and economic resources if their parents break up, which is an argument that could also serve to explain why children belonging to ethnic and racial minorities experience fewer negative consequences from parental separation (see McLanahan & Sandefur, 1994, for the US; Kalmijn, 2010, for the Netherlands).

Based on existing studies on socioeconomic heterogeneity in the non-intact penalty, the overarching research question of this paper is whether, and in which direction, the migration background moderates the relation between living in a non-intact family and children’s educational outcomes. The potential moderating role of the migration background has been somewhat neglected within the literature (Erman & Härkönen, 2017), especially for the Italian case, which represents the focus of this paper. In fact, to the best of our knowledge, this study is the first to analyse the differences in the non-intact penalty between native and migrant children in Italy.

Italy is a relatively ‘new’ destination for international migrants from many different areas of origin, including countries with little or no previous economic, political, or cultural ties. Migration inflows increased in the early 1990s, with an initial prevalence of male migration from Morocco and Albania, and intensified substantially in the 2000s, especially from Romania, China, and ex-Soviet countries (e.g. Ukraine and Moldova)*.* Different from prior US-based research—which focused on black/white differences—it is particularly important to investigate the possible heterogeneity in the non-intact penalty in terms of migration background, that is: comparing the non-intact penalty among natives, first-, and second-generation migrant children from different areas of origin. While there is ample evidence to suggest educational achievement gaps between immigrant and native pupils in Italy (Azzolini et al., 2012; Triventi et al., 2021), the interaction with family structure has yet to be explored. We do so by means of data from the ‘Integration of the Second Generation’ survey, conducted by the Italian National Institute of Statistics (ISTAT) in 2015. Such data have been rarely used in studying the topic so far, despite their representing a unique source of information on migrant-background children in Italy while also surveying a comparable number of native-background children. We analyse the association between living in a single-parent household and children’s lower secondary school grades, as well as upper secondary school aspirations. We explore native–migrant differences in the association, distinguishing migrant children on the basis of their generational status (first and second generation) and area of origin (Eastern Europe; East-South-and-Southeast Asia; Middle East and North Africa; Else).

We also explore the mechanisms underlying possible differences in the non-intact penalty between natives and migrants, considering their socioeconomic resources and family environment. Moreover, the present study does not overlook the notion that reasons for parental absence are not the same for native and migrant children. For the latter, so-called transnational households come into play. Transnational households’ members are spatially dispersed across different nations, often for long periods of time, but they remain together in such a way as to preserve the feeling of unity, and the continuation of reciprocity and obligations across borders (Bryceson & Vuorela, 2020; Parreñas, 2005). For migrant households, separation can be seen as part of a strategy conceived for improving the social mobility of all members without necessarily dissolving the family itself (González-Ferrer et al., 2012). Consequently, the implications of parental absence related to transnational practices can differ from those caused by breakups.

The remainder of the paper is structured as follows. The next section reviews the extant literature on the association between non-intact families and children’s educational outcomes, with particular attention paid to the mechanisms leading to heterogeneity in terms of parental SES and ethnic background. This literature is then applied to the study of migrant–native differences in the association, focusing on the Italian case. Next, we describe the data employed, the operationalisation of variables, and the plan of analysis. Finally, we report the study’s main results and draw our final conclusions.

## Theoretical Background

### Non-intact Families and Children’s Educational Outcomes: Heterogeneity and Mechanisms at Play

For both Europe and the US, there is ample empirical evidence to suggest that children of divorce and separation, or those living with a single parent at birth, fare worse in terms of educational outcomes than children living with both parents (Amato, 2000, 2010; Guetto et al., 2022)—what we refer to as the ‘non-intact penalty’. Recent research in this field has followed two main lines of inquiry. First, some studies have sought to understand whether the negative association between single-parent households and children’s educational outcomes is causal or due to selection processes. The consensus is that, although smaller in magnitude, the negative effects of parental divorce/separation and absence of the father on children’s outcomes remain even after controlling for unobserved family characteristics (Amato, 2010; Amato & Anthony, 2014). The second line of research has focused on heterogeneity in the non-intact penalty. Differences across social groups defined by SES and ethnicity have recently captured the attention of researchers (Aquino et al., 2022). In what follows, we summarise the main findings of the second stream of research, which provides the theoretical and empirical foundations for our analysis of the heterogeneity in non-intact penalties by migration background.

Sociologists have recently tried to uncover whether parental separation has a stronger effect on the educational outcomes of children from high- or low-SES families (Bernardi & Boertien, 2017). Two conflicting expectations can be advanced in this regard. On the one hand, high-SES families—by means of all of the social, cultural, and economic resources available to them—might be better able to shield their children from the negative consequences of separation, which could be seen as an example of the compensatory advantage mechanism (Bernardi, 2014). On the other hand, it has been argued that children from high-SES families might suffer more negative consequences than those from low-SES families precisely because they have more social, cultural, and economic resources to lose if their parents break up (Bernardi & Boertien, 2016). Either way, low-SES parents have fewer resources to invest in their children’s education, which leads to lower educational outcomes compared to children of high-SES families, regardless of family disruptions. Such a ‘more to lose’ mechanism may also be driven by a floor effect. For instance, the chances of reaching tertiary education are already so small for children of low-educated parents that parental separation has limited room to affect them (Bernardi & Radl, 2014). The available empirical evidence is conflicting and seems to point in both directions (see, e.g. Amato & Anthony, 2014; Grätz, 2015 vs Bernardi & Radl, 2014; Nilsen et al., 2020). The differences in the results could be due to several factors, including methodological and operational choices, as well as the type of educational outcome considered (Bernardi & Boertien, 2017; Guetto & Panichella, 2019).

The ‘more to lose’ mechanism seems particularly apt for explaining the well-known finding that the effects of parental separation are weaker among black children in the US than in their white counterparts (McLanahan & Sandefur, 1994). There is ample empirical evidence to suggest that standardised household income declines considerably following parental separation (Amato, 2010; Ongaro et al., 2009), and children living in single-parent households—usually with a lone mother—are exposed to much higher risks of poverty (Aassve et al., 2007; Thévenon et al., 2018). In the light of the higher unemployment rates and lower income in black US households (Thiede et al., 2017), parental separation should imply a higher loss of economic resources for white children, which should translate into more negative consequences for their educational attainment. Although ethnic and racial differences in non-intact penalties have been less frequently explored in Europe, Kalmijn (2010) found a similar result when comparing Caribbean and white Dutch children in the Netherlands.

Another reason why living in a single-parent household may be less detrimental for black/minority children than white/majority ones concerns differences in the diffusion and ‘acceptance’ of divorce. The idea is that, since divorce is more common—and thus more institutionalised—among certain ethnic and racial groups (e.g. among black Americans, Raley & Sweeney, 2020), children belonging to these groups experience less social stigmatisation, with fewer negative consequences for their well-being (Kalmijn, 2010).

### Heterogeneity in the Effects of Single-Parent Households by Migration Background

In the following paragraphs, we extend the abovementioned discussion on heterogeneity in the non-intact penalty, and its underlying mechanisms, to the analysis of possible differences by migration background.

The ‘more to lose’ argument points towards a more detrimental effect of living in a single-parent household for the educational outcomes of natives than migrant’s children in Western societies. A growing body of literature has highlighted the systematic and substantial disadvantage experienced by ethnic minorities in Western European labour markets, in terms of both employment chances and occupational attainment (Heath & Cheung, 2007). Although the socioeconomic disadvantage of immigrants takes different forms across European countries, with regard to its magnitude and characteristics (Fullin & Reyneri, 2011), lower parental socioeconomic status represents an important source of migrant–native educational gaps in most Western European societies (Heath & Brinbaum, 2007). In the light of these stylised facts, one may argue that parental breakups should be less relevant for migrant children compared to their native counterparts as the latter have more economic resources to lose in cases of family disruption. Migrant children’s educational outcomes are also hampered by sociocultural factors, such as difficulties in acquiring the host-country language (Kristen, 2019) and teachers’ discrimination (Sprietsma, 2013), both of which contribute to their lower educational performances, regardless of the type of family arrangement in which they live.

Migrant children’s schooling experiences show remarkable generational and country of origin variations. It has been well-established that native-born children of migrants (the second generation) tend to outperform foreign-born children (the first generation) (Heath et al., 2008). Second-generation migrant children do not directly face the hurdles of migration and the difficulties of adapting to new contexts, languages, and schools. However, this adaptation process may not be equal across country of origin groups. The literature concerning Western Europe has highlighted a stronger, and inter-generationally persistent, educational disadvantage for children from Muslim-majority countries due to the stronger labour market disadvantage of their parents, and higher cultural and language barriers (Heath & Brinbaum, 2007). Variations in migrant children’s educational outcomes may also imply differences in the non-intact penalty across migrant generations and countries of origin. Second-generation migrants and children originating from countries that are culturally and economically closer to the destination show educational outcomes similar to those of natives (Heath et al., 2008). Thus, as per the ‘more to lose’ argument, they should be more vulnerable to the negative consequences of parental breakups and living in a single-parent household than first-generation migrants and children belonging to more culturally distant and economically disadvantaged minorities.

Two recent studies have found large differences in family structures across migrant groups in Western European societies, even after controlling for their socioeconomic composition. In line with previous research into comparisons between black and white children in the US, these studies have found weaker negative effects of parental separation and father absence on children’s well-being and educational outcomes in migrant groups where non-intact families are more common (Erman & Härkönen, 2017; Kalmijn, 2010). These results suggest that, even among migrant groups in Western Europe, non-intact penalties seem dependent on differences in the degree of acceptance and institutionalisation of separation and divorce.

### Migrant Children in Single-Parent Households: The Role of Transnational Families

Much of the literature discussed in the previous paragraphs concerns heterogeneity in the effects of family disruptions following separation or divorce. However, the absence of one parent could be due to different family dynamics in native and migrant families. Whereas separations and divorces represent by far the most common reasons for native children to live with a single parent in contemporary Western societies, (temporary) parental separation among migrant children can be part of the family’s migration strategy.

Over the past twenty years, transnational family studies have examined the effects of family disruptions, with a primary focus on the migrant parent(s) and their children who remained in the origin country. Many authors have suggested that living separately from their children is part of a life plan for transnational migrants who left to seek more favourable employment opportunities abroad (Schmalzbauer, 2004; Zontini, 2004). Thus, both mothers and fathers accept the material and emotional sacrifices generated by migration for the purpose of improving their transnational family’s living standards. In other words, diversifying family resources and income to reduce the risk of poverty and provide for the household is the strategic goal of this type of migration (Goldin & Reinert, 2012). After an initial period of distress, children who experience the spatial separation from one or both parents adapt to the new living arrangements and dynamics. The majority of these children experience relatively modest and short-lived effects of parental absence, depending on the quality of the long-distance parenthood. Generally speaking, trying to preserve strong intra-family relationships across international borders and redefining the roles between family members represent crucial components of migrants’ lives in transnational families (Fresnoza‐Flot^2009^; Parreñas, 2005). Thus, for children, the geographical separation from one or both parents can be compensated by the preservation of strong ties with them, and by the quality of the childcare that is entrusted to other relatives. Likewise, these factors allow migrant mothers and fathers to participate in raising and educating their children from a distance (Hondagneu-Sotelo & Avila, 1997; Parreñas, 2001).

Accordingly, parent–child separations caused by divorce and migration represent two distinct experiences, each with different possible effects. Scholars have found that migrant fathers who live apart from their children often remain more actively engaged in their lives than their counterparts who live separated after divorce (Dreby, 2010; Nobles, 2011), which may translate into different effects on children’s educational outcomes. In fact, empirical studies on the schooling of ‘left-behind’ children found mixed results. Children of migrants who have been left behind may be equally, or even better, educated than children of non-migrant parents, when remittances are used to invest in children’s schooling (Curran et al., 2001; Goldin & Reinert, 2012). However, other studies have suggested that prolonged parent–child separation can influence the quality of parenthood and might be negatively associated with children’s scholastic success (Giannelli & Mangiavacchi, 2010; McKenzie & Rapoport, 2011).

As most of the research on transnationalism has focused on the migrant parents and their left-behind children, the implications of parent–child separation on first- and second-generation children in the destination country have been largely neglected. The emerging literature on this topic suggests that children of immigrants in transnational families, but born or raised in host countries, experience daily interactions with their parents and relatives across borders, and consequently often adapt to their transnational family arrangement (Fokkema et al., 2012; Lee, 2011). At the same time, the possible negative effects of parent–child separation can be mitigated by a higher level of economic and sociocultural integration (Klok et al., 2020).

### The Italian Setting

While Italy has become an important destination for international migration since the early 1990s, migration inflows substantially intensified during the 2000s following the European Union Eastern Enlargements (Azzolini et al., 2017). Two main features of migration inflows towards Italy are worth highlighting. First, compared to other Western European countries, Italy has a much higher prevalence of unauthorised economic migrants who arrive without a job and often become involved in the underground economy (Reyneri, 2016). Second, the feminisation of the more recent migration waves, which have included many women from Latin American and, especially, Eastern European countries migrating alone, is also a factor worth considering. As they often leave their families in the country of origin, transnationalism is an important feature of their migratory project (Ambrosini, 2015).

The ‘more to lose’ mechanism may be especially relevant for explaining migrant–native differences in the non-intact penalty in Italy. Studies on the socioeconomic integration of immigrants in Italy have indeed described a pattern of strong disadvantage. Compared to immigrants in Central and Northern European countries, those in Italy tend to find jobs with relative ease, albeit at the cost of being more strongly segregated in the secondary labour market, which is characterised by poorer working and economic conditions (Fullin & Reyneri, 2011). Immigrants with previous labour market experience in their origin countries face strong occupational downgrades on arrival, followed by very scant opportunities of upward social mobility (Fellini & Guetto, 2019). These dynamics translate into high poverty risks for migrant families. In 2019, 31.2% of families of foreign citizenship with minor children were in absolute poverty, compared to 6.3% among their Italian counterparts (ISTAT, 2019). Accordingly, very large educational gaps between migrant and native pupils have been identified in Italy (Azzolini & Barone, 2013; Schnell & Azzolini, 2015). While such gaps are smaller among the second generations, and especially among mixed-parentage children (Azzolini et al., 2017), they remain substantial even after controlling for parental SES and language skills (Azzolini et al., 2012; Triventi et al., 2021). However, the international literature suggests that some origin groups that are severely disadvantaged in the first generation (e.g. East Asians) completely catch up with (and often outperform) natives in the second generation, while migrant children from Muslim-majority countries seem to lag behind even when born in Italy (Azzolini & Barone, 2013).

In the light of the poor socioeconomic integration of immigrants and the existence of strong migrant–native educational gaps, living in a non-intact family may be less consequential for migrant children’s educational outcomes in Italy compared to those of native children—especially when focusing on the first generation and more disadvantaged migrant groups. Migrant–native differences in non-intact penalties in Italy may not necessarily have their origins in the differing degree of acceptance of divorce across groups. The most recent immigration waves contained migrants from countries where divorce rates are comparable to the Italian one (e.g. Romania, Albania). Children originating from Eastern Europe and Latin America may show a higher probability of living in a single-parent household compared to those of Arab and sub-Saharan descent, but this is likely due to the higher prevalence of transnationalism in the former migrant groups.

## Research Hypotheses

This study explores differences in the non-intact penalty between native and migrant children, taking into consideration the variations of generation and country of origin, as well as differences in the reasons behind parental absence. Adopting a ‘more to lose’ perspective, the difficult socioeconomic integration of immigrants and the existence of substantial migrant–native educational gaps suggest stronger negative effects of family disruptions for natives, which leads to our first hypothesis:

### H1

Non-intact penalties are stronger for native than for migrant children.

However, migrant–native differences in non-intact penalties might differ based on migrant children’s generational status. In fact, the ‘more to lose’ mechanism could well be more relevant when comparing natives and first-generation migrant children. The latter, especially if arriving in the host country during their early teens, are particularly disadvantaged in terms of schooling due to their experiencing the double disruption of migrating and having to adapt to a new context, language, and school environment. Second-generation migrant children may instead be more similar to their native peers, and thus more exposed to the negative consequences of parental separation. Accordingly:

### H2a

Non-intact penalties are stronger for second- than for first-generation migrants.

We do not expect strong heterogeneity in non-intact penalties across areas of origin. Theoretical predictions based on the ‘more to lose’ mechanism and the degree of diffusion and acceptance of divorce seem to work in the opposite direction in the Italian context. On the one hand, children from Muslim-majority countries have been found to experience larger migrant–native educational gaps compared to other migrant groups in Italy (which would imply lower non-intact penalties). On the other hand, the same migrant group usually shows a lower incidence of single-parent families (which would imply higher non-intact penalties). This leads us to hypothesis H2b:

### H2b

Non-intact penalties show limited heterogeneity based on migrant children’s area of origin.

We also seek to disentangle the mechanisms underlying non-intact penalties and their heterogeneity by migration background. Based on the literature on the consequences of parental separation, we formulated our third hypothesis:

### H3

Stronger non-intact penalties for native children can be explained by the loss of economic resources and the worsening of the family environment.

When analysing heterogeneity by migration background in non-intact penalties, the reason for parental absence should also be considered. In native families, parental absence is mostly the result of separation or divorce, with the related issues of family conflict, stress, and reduced parent–child contact—especially those with the father—all factors that are usually associated with negative consequences for children’s educational outcomes. However, among migrant children, parental absence is often related to transnational family arrangements, which may be less disruptive and have more ambiguous consequences for their schooling. The higher incidence of transnationalism among migrant families may contribute to explaining the weaker non-intact penalties experienced by migrant children. This leads us to our fourth hypothesis:

### H4

Separation is associated with stronger non-intact penalties compared to transnationalism, and this accounts for at least part of the weaker penalties found for migrant children.

## Data, Variables, and Methods

### Data

We employed data from the 2015 ‘Integration of the Second Generation’ (ISG) survey, conducted by the Italian National Institute of Statistics (ISTAT). It involved 68,127 Italian and non-Italian children attending lower and upper secondary schools, and collected information on several life domains, including their migration background, school performance, living arrangement, and socioeconomic situation. The survey was based on a sample of 1400 secondary schools located in 821 Italian municipalities, attended by at least 5 non-Italian children (immigrant or foreign status being defined based on citizenship). Italian children were sampled as the control group. Therefore, in each class, the same number of non-Italian children were eligible for the survey.

In the Italian educational system, lower secondary education (ISCED 2) is an undifferentiated track lasting three years, and attended by all children aged roughly 11–13. As long as no school years must be repeated, the children enter upper secondary school at age 14. Upper secondary education is comprised of three tracks: the general and academically oriented, the technically oriented, and the vocational track.

The children completed an online questionnaire (CAWI) during school hours and were supported by a trained interviewer if needed. The data collection took place between March and June 2015. Roughly 98% of the sampled schools participated in the survey; among those, only 2.5% did not employ the CAWI technique for technical problems, opting instead for a traditional pen-and-paper personal interview (PAPI) method. Among non-Italian pupils, the response-rate was 82.1%; the response-rate for Italian pupils could not be calculated due to their being interviewed to serve as the control group.

### Analytical Sample

We selected all Italian and non-Italian children attending lower secondary school (*N* = 32,700). We applied this restriction because the survey reported no information on the track attended by children in upper secondary school. In fact, school grades cannot be compared across tracks, and track choice is highly stratified in terms of migration and socioeconomic background (Azzolini & Barone, 2013). Therefore, including upper secondary school children without being able to control for school type could have led to biased results. Moreover, we excluded children living without either of their biological parents, as they represented a minority (*N* = 338) and their situation might strongly differ from those living in single-parent households. Similarly, foreign-born children born in Western Europe, North America, South Africa, Japan, Australia, and New Zealand were also excluded from the sample (*N* = 417). After further excluding records with missing information on the dependent variables (*N* = 899), our analytical sample amounted to 31,046 pupils.

### Variables

The first dependent variable was the *grade in mathematics* obtained in the last school report, which would have been few weeks before the interview took place for most pupils.^1^ It ranged between 0 (the lowest) and 10 (the highest), with 6 representing the passing grade. We opted for mathematics grades as these tend to be less affected by language skills.

The second dependent variable was *upper secondary school aspirations*, which refers to the school track children reported wishing to attend after completing lower secondary school. The variable had the following five categories: the most prestigious academic tracks (*liceo classico* or *scientifico*); technical schools (*istituto tecnico*) and less prestigious academic tracks (*liceo linguistico, pedagogico,* or *artistico*); vocational schools (*istituto professionale*) and short professional courses; ‘else’; and ‘don’t know’ for children who did not answer the question, possibly because they had no clear aspirations as yet. In the Italian educational system, the choice of the upper secondary track is up to children and their families, and is independent from previous grades and teachers’ recommendations. Thus, such family characteristics as parental education (Guetto & Vergolini, 2017) and family structure (Guetto & Panichella, 2019) have been found to play a highly significant role for upper secondary school decisions in Italy. Considering this second educational outcome is relevant because the choice of the upper secondary track impacts children’s future life courses and opportunities. Whereas the majority of children who attend the most prestigious academic tracks subsequently enrol in university, only a small share of those who attend the other tracks (and especially more vocationally oriented courses) do so (Ballarino & Panichella, 2016).

The first independent variable operationalised family structure. In the first formulation, we identified children living in a *non-intact family* at the time of the interview,^2^ with a dummy taking the value of 1 if either the mother (*N* = 456) or father (*N* = 4595) was absent, and 0 when both parents were present in the household. In the second formulation, we distinguished four *types of non-intact family*: non-intact due to separation (the absent parent lives either in the same city but in another house, or in a different Italian city), transnationalism (the absent parent lives abroad), death (the absent parent is deceased), and a residual category for a minority of children (less than 2% of the sample) who did not know where the absent parent lives (the results of whom are not shown in the paper). It is important to stress that the data did not allow us to identify the exact reason for parental absence, meaning that we could only infer this indirectly. Whereas, for native children, it is relatively safe to assume that, in most cases, union dissolution is the reason behind the absence of a living parent, ‘transnationalism’ does not rule out the possibility that migrant children have also experienced parental breakup. We will return to this issue when discussing our results.

The second independent variable related to *migration background*, and measured *immigrant generations* according to the combination of children and parents’ place of birth (Italy vs abroad). Children were classified into the following categories: (i) Italian-born children of at least one Italian-born parent (*natives*); (ii) Italian-born children of two foreign-born parents, the so-called second generation (*2-Gen*); and (iii) Foreign-born children of at least one foreign-born parent (*1-Gen*). The native group included mixed-parentage children born in Italy (*N* = 1649), as a sensitivity analysis showed them to be virtually indistinguishable from children with two Italian-born parents in terms of non-intact penalties. Furthermore, we included foreign-born children of two Italian-born parents (*N* = 413) in the first group: considering these children’s countries of birth—mostly Eastern and Western European countries—they were either adoptees or children of return migrants. We checked to see whether eliminating this group would leave our results unchanged. The *1-Gen* group included immigrant generations from 1.25 to 1.75 (Rumbaut, 2004),^3^ and was also comprised of foreign-born children of mixed-unions (*N* = 546). We did so after verifying that these children were virtually indistinguishable, in terms of non-intact penalties, from foreign-born children with two foreign-born parents.

The third independent variable also related to *migration background*, but distinguished migrant children by their *area of origin.* For *1-Gen* children, we took their area of birth into account, while for *2-Gen* children, we considered their mothers’ or, if unavailable, their fathers’ area of birth. This distinguished native children, as defined above, from children with the following origins: Eastern Europe (*EastEU*, 40% from Romania and 28% from Albania); South, East, and Southeast Asia (*S/E/SEAsia*, 40% from China and 23% from the Philippines); Middle East and North Africa (*MENA*, 67% from Morocco and 15% from Tunisia); and a residual category (*Else*) in which we aggregated several countries from Latin America and Sub-Saharan Africa, whose sample size was too small to form separate groups.

We considered three sets of control variables. The first included *socio-demographic characteristics*: sex; age (10–15+); school grade (1st, 2nd, and 3rd year: *prima, seconda,* or *terza media*); number of co-resident siblings (0–5+); macro-area of the school (North-West, North-East, Centre, South, Islands); mother and father’s educational level (no title and elementary school; lower secondary; upper secondary; university and more; don’t know). The second set included variables concerning the *economic condition of the household* in which children live: the self-reported economic condition of the family (quite/very rich; neither rich nor poor; quite/very poor); a variable counting the number of objects or appliances belonging to the household (dishwasher, computer, DVD player, car, motorbike); a four-category variable for the number of people per room (PPR), obtained dividing the number of co-resident individuals by the number of rooms in the house (< 0.76, between 0.76 and 1.24, > 1.24, and an additional category for children in non-conventional housing, such as group homes); finally, a dummy variable with a value of 1 if the household has no one to rely upon in case of need. The third set of controls concerned measures of the *family environment*. We built four indexes (ranging between 0 and 5) with an exploratory factor analysis. Higher values indicated a better family environment and parent–child relationship: an index for parents’ involvement with children’s schooling (*school involvement*), based on two items concerning whether the child often shares what happens at school with their parents, and whether the parents ask about the child’s school performance; a *parenting quality* index considering whether family members help each other, are sensitive to the child’s feelings and needs, are respectful and encouraging towards the child’s opinions, and calmly explain the child’s mistakes; a *punishment* index, which captured whether family members have frequent arguments, whether parents administer punishments without explanations, and harsh reprimands for mistakes; finally, an *indifference* index composed of two items on whether family members are indifferent to the child’s mistakes, and hold back and do not scold the child even in cases of disruptive or subversive behaviour.

Descriptive statistics are reported in Tables 2 and 3 (‘Descriptive Results’ section), and Table 4 (Appendix).

### Methods

To test H1 and H2a, we modelled grades in mathematics by means of OLS regression models, with migration background (*Native*, *2-Gen*, *1-Gen*) and the dummy for non-intact family, and their interactions, included as the main independent variables. To test H2b, we implemented OLS models where we measured migration background as distinguished by both generational status and area of origin.

Adopting a stepwise logic, the models first included only the socio-demographic control variables (Step 1). We then augmented them with the economic condition variables (Step 2). Finally, we included controls for family environment (Step 3). The variables included in Steps 2 and 3 may be considered as *intervenient*, since they are usually affected by changes in family structure, and thus may account for part of the non-intact penalties (H3).

To test H4, we distinguished between non-intact families by drawing on the reason for parental absence (separation, transnationalism, death, else). Therefore, school grades were regressed on the categorical non-intact variable in interaction with migration background (*Native*, *2-Gen*, *1-Gen*). Due to issues related to sample size and the limited differences in the non-intact penalty across origin areas, we defined migration background based only on generational status. In this case, we added control variables as in Step 1 so as to grasp any potential differences in the *total* effects of different types of non-intact families.

Finally, we also tested H1, H2a, and H3 on a different educational outcome, namely upper secondary school aspirations, which we explored through multinomial logistic regression models. We used the dichotomous non-intact variable in interaction with migration background (*Native*, *2-Gen*, *1-Gen*) as the main independent variables, and added control variables in a stepwise manner. All of the results are presented in graphical form, but full models are available as supplementary materials (Tables S1 to S4).

While we applied sampling weights to all regression models, we checked that the results remained virtually the same without them. Our analysis plan is summarised in Table 1.

**Table 1:** Analysis plan

X1	X2	Y	Method	Controls
Non-intact (binary)	Migration background (generation)	School grades (0–10)	OLS (X1#X2)	Step 1, 2, 3
Non-intact (binary)	Migration background (area of origin & generation)	School grades (0–10)	OLS (X1#X2)	Step 1, 3 (*2 not shown)*
Non-intact (family types)	Migration background (generation)	School grades (0–10)	OLS (X1#X2)	Step 1
Non-intact (binary)	Migration background (generation)	Upp. sec. school aspirations	Mlogit (X1#X2)	Step 1, 3 (*2 not shown)*

## Results

### Descriptive Results

Table 2 shows the descriptive statistics of the dependent and independent variables. Consistent with our expectations, natives achieved higher grades in mathematics than migrants, with *1-Gen* children being slightly more disadvantaged than *2-Gen* children. In terms of upper secondary track aspirations, whereas 30% of natives aimed at the most prestigious academic tracks, only 25% and 19% for *2-Gen* and *1-Gen* children, respectively, also did so. We found native and *2-Gen* children to have the same likelihood of living in a non-intact family during lower secondary school (13%). The higher incidence of non-intact families among *1-Gen* migrants (25%) could well be the result of the higher share of transnational families (14%), which was less common among *2-Gen* children (4%) and extremely rare among natives (< 1%).

**Table 2:** Descriptive statistics, by migration background (generation)

	Natives		2-Gen		1-Gen		Total	
	Mean; N	SD; *%*	Mean; N	SD; *%*	Mean; N	SD; *%*	Mean; N	SD; *%*
*Dependent variables*								
Mathematics grades	7.09	1.15	6.58	1.10	6.42	1.10	6.83	1.17
Upper secondary school aspirations								
Academic track	5316	*30.33*	1379	*24.81*	1541	*19.36*	8236	*26.53*
Technical, lower academic	6939	*39.59*	2125	*38.23*	2886	*36.26*	11,950	*38.49*
Vocational	2604	*14.86*	954	*17.16*	1780	*22.36*	5338	*17.19*
Else	1384	*7.9*	515	*9.27*	784	*9.85*	2683	*8.64*
Don’t know	1286	*7.34*	585	*10.53*	968	*12.16*	2839	*9.14*
*Independent variables*								
Family structure								
Intact family	15,193	*86.67*	4814	*86.61*	5988	*75.24*	25,995	*83.73*
Non-intact family	2336	*13.33*	744	*13.39*	1971	*24.76*	5051	*16.27*
Types of non-intact family								
Transnational	115	*0.66*	234	*4.21*	1133	*14.24*	1482	*4.77*
Separation	1794	*10.23*	328	*5.9*	376	*4.72*	2498	*8.05*
Death	281	*1.6*	80	*1.44*	200	*2.51*	561	*1.81*
Don’t know	146	*0.83*	102	*1.84*	262	*3.29*	510	*1.64*
Total	17,529	*100*	5558	*100*	7959	*100*	31,046	*100*

Mean and standard deviation (SD) are reported for continuous variables; cell size (N) and column percentages (*%*, in italics) are reported for categorical variables. *N* = 31,046. Unweighted data. Source: Integration of the Second Generation survey (ISTAT 2015)

Comparing children by area of origin (Table 3), the share of *2-Gen* children in non-intact families was approximately 10% in the *EastEU*, *MENA*, and *S/E/SE Asian* origin groups. In general, the reasons for living in a non-intact family were similarly distributed among these migrant groups (e.g. separation occurred in 3–5% of families among both *2-* and *1-Gen* children). Transnationalism was generally more common among *1-Gen* children from Eastern Europe, and especially in the residual category *Else* with many of these children originating from more geographically distant countries in Latin America and Sub-Saharan Africa. These descriptive results suggest that separations and divorces are similarly distributed among groups, and generally not more common among ethnic minorities compared to Italy’s majority population.

**Table 3:** Descriptive statistics: family structure by migration background (area of origin and generation)

Area of origin	Intact	Types of non-intact family				Total
		Transnational	Separation	Death	Else	
*Native* (N)	15,193	115	1794	281	146	17,529
*%*	*86.67*	*0.66*	*10.23*	*1.6*	*0.83*	*100*
*2-Gen*						
EastEU (N)	2243	63	121	34	38	2499
* %*	*89.76*	*2.52*	*4.84*	*1.36*	*1.52*	*100*
S/E/SEAsia (N)	972	21	49	13	14	1069
* %*	*90.93*	*1.96*	*4.58*	*1.22*	*1.31*	*100*
MENA (N)	964	49	43	14	17	1087
* %*	*88.68*	*4.51*	*3.96*	*1.29*	*1.56*	*100*
Else (N)	635	101	115	19	33	903
* %*	*70.32*	*11.18*	*12.74*	*2.1*	*3.65*	*100*
*1-Gen*						
EastEU (N)	3608	711	233	131	204	4887
* %*	*73.83*	*14.55*	*4.77*	*2.68*	*4.17*	*100*
S/E/SEAsia (N)	897	51	53	10	13	1024
* %*	*87.6*	*4.98*	*5.18*	*0.98*	*1.27*	*100*
MENA (N)	730	64	25	18	3	840
* %*	*86.9*	*7.62*	*2.98*	*2.14*	*0.36*	*100*
Else (N)	753	307	65	41	42	1208
* %*	*62.33*	*25.41*	*5.38*	*3.39*	*3.48*	*100*
Total (N)	25,995	1482	2498	561	510	31,046
*%*	*83.73*	*4.77*	*8.05*	*1.81*	*1.64*	*100*

Cell size (N) and row percentages (*%*, in italics) are reported. *N* = 31,046. Unweighted data. Source: Integration of the Second Generation survey (ISTAT 2015)

Due to space limitations, descriptive statistics concerning control variables are reported in Table 4 of the Appendix. We found natives to be twice as likely to have a tertiary educated mother than *2-* and *1-Gen* children (20% vs 10%), as well as more likely to have a tertiary educated father (16% vs 9% among *2-* and *1-Gen* children), which likely accounts for at least part of the migrant–native grade gap. However, it is important to highlight the high percentages of cases in the ‘don’t know’ category, ranging from 25% for natives’ mother’s education to 37% for *2-Gen* father’s education.

### Non-intact Family and School Grades: Differences by Migrant Generations

**Fig. 1:**
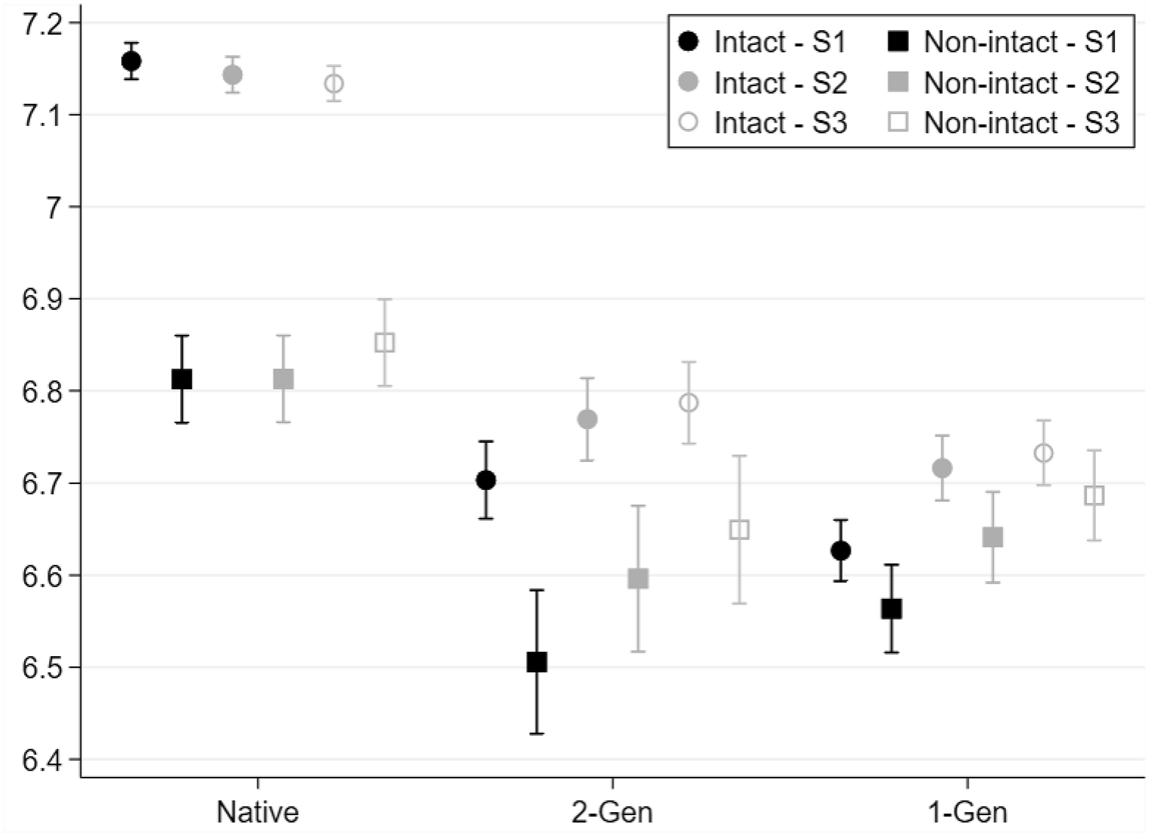
School report grades (mathematics), by family structure and migration background (generation). *Note* Adjusted predictions after OLS models, with 83.5% C.I. Models control for socio-demographic characteristics (Step 1) and are augmented with the economic condition of the household (Step 2) and family environment (Step 3). *N* = 31,046. Source: Integration of the Second Generation survey (ISTAT 2015)

Figure 1 shows predicted grades in mathematics for children attending lower secondary school, by family structure—‘intact’ (circles) vs ‘non-intact’ (squares)—and migration background, adjusted for different control variables according to the model’s steps.^4^ Even after controlling for socio-demographic characteristics (Step 1, black symbols), native children reported higher grades in their last school reports than their *2-Gen* and *1-Gen* counterparts. However, whereas native children living in intact families scored roughly 7.2 on average, those living in non-intact families had an average grade of 6.8. Among *2-Gen* children, the non-intact penalty was smaller (average grades of 6.7 and 6.5), although statistically significant at the 5% level. Among *1-Gen* children, the non-intact penalty was almost non-existent.^5^ These initial results confirm H1 and H2a, and suggest that the non-intact penalty somehow ‘equalises down’ school performances, even if native children from non-intact families still outperform migrant children living in intact families. Additional analyses concerning grades in Italian (Fig. 5 in Appendix) show remarkably similar results, but the non-intact penalty was slightly larger for natives (roughly 5 decimal points) and *2-Gen* children (approximately 4 decimal points).

In Step 2 (grey symbols, Fig. 1), the models are augmented with variables concerning the household’s economic condition. Whereas migrant–native educational gaps were partly reduced under this model (for *1-Gen* children in particular), non-intact penalties for native and *2-Gen* children remained largely unchanged. This result suggests that native–migrant children's differences in school performance are partly due to differences in their socioeconomic backgrounds. However, the stronger non-intact penalty for native children cannot be attributed to the worse economic conditions of their single-parent households. In Step 3 (white symbols, Fig. 1), predicted grades were also adjusted for variables related to the family environment. The inclusion of these variables further contributes to explaining migrant–native educational gaps, although to a more limited extent; in this case, the non-intact penalty for native and *2-Gen* children also appeared to be slightly reduced. In sum, although the results are consistent with the ‘more to lose’ mechanism, they provide only limited support to H3, which stated that stronger non-intact penalties for native children could be explained by the loss of economic resources and a worsening family environment. In fact, shifting from Step 1 to Step 3, the non-intact penalty for native children was only reduced by approximately 20%, remaining of almost 3 decimal points.

Additional results (based on a multinomial logistic regression, see Fig. 6 in Appendix) suggest that it is very unlikely for children in lower secondary school to obtain a failing grade in mathematics in the school report, regardless of their migration background. However, native children from intact families were far more likely to obtain excellent grades (8+) than their *1-* and *2-Gen* counterparts. The non-intact penalty for native children found in Fig. 1 is thus largely due to a much lower probability of obtaining excellent grades, and a higher probability of obtaining passing grades (6).

### Non-intact Family and School Grades: Differences by Area of Origin

**Fig. 2:**
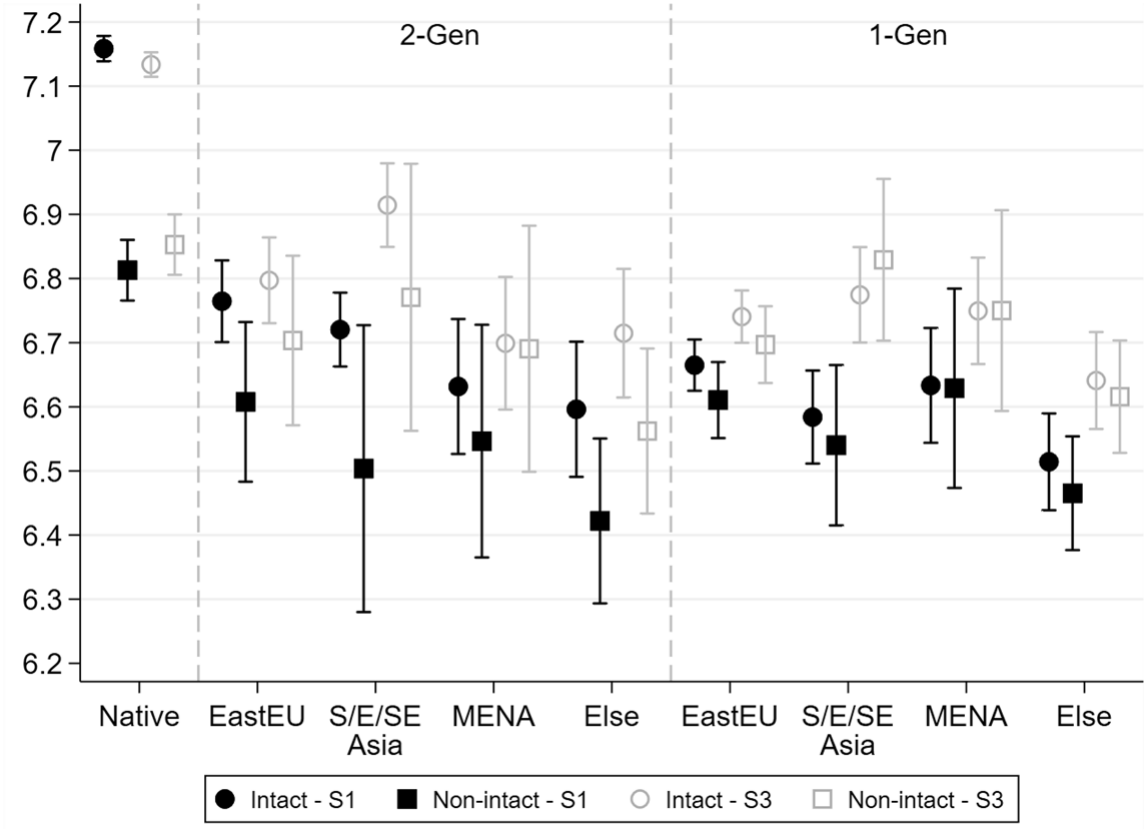
School report grades (mathematics), by family structure and migration background (area of origin & generation). *Note* Adjusted predictions after OLS models, with 83.5% C.I. Models control for socio-demographic characteristics (Step 1) and are augmented with the economic condition of the household (Step 2, not shown) and family environment (Step 3). *N* = 31,046. Source: Integration of the Second Generation survey (ISTAT 2015)

Figure 2 shows the non-intact penalty when distinguishing immigrant generations by area of origin.

The results show very limited heterogeneity in the effects of living in a non-intact family by areas of origin. The small, but statistically significant, non-intact penalty observed for *2-Gen* children in Fig. 1 is not driven by a single immigrant group, but is rather confirmed across all areas of origin, notwithstanding the much higher estimation uncertainty due to the smaller sample sizes. Similarly, Fig. 2 shows no penalties for *1-Gen* children regardless of their area of origin. These results are consistent with H2b, and suggest that ethnic-specific mechanisms, such as the different diffusion and acceptance of separation and divorce across origin groups, do not play a substantial role for the size of the non-intact penalty in Italy—or, as we hypothesised, that they would offset each other.

However, this should not be taken to mean that there are no differences across immigrant groups in the patterns of educational disadvantage. For both *2-* and *1-Gen* children from South, East, and Southeast Asia (*S/E/SE Asia*), the educational disadvantage shrank substantially once controlling for the mediating variables (Step 3), whereas other groups lagged behind, a result consistent with previous evidence for Italy (Azzolini & Barone, 2013).

### Non-intact Family Types and School Grades

**Fig. 3:**
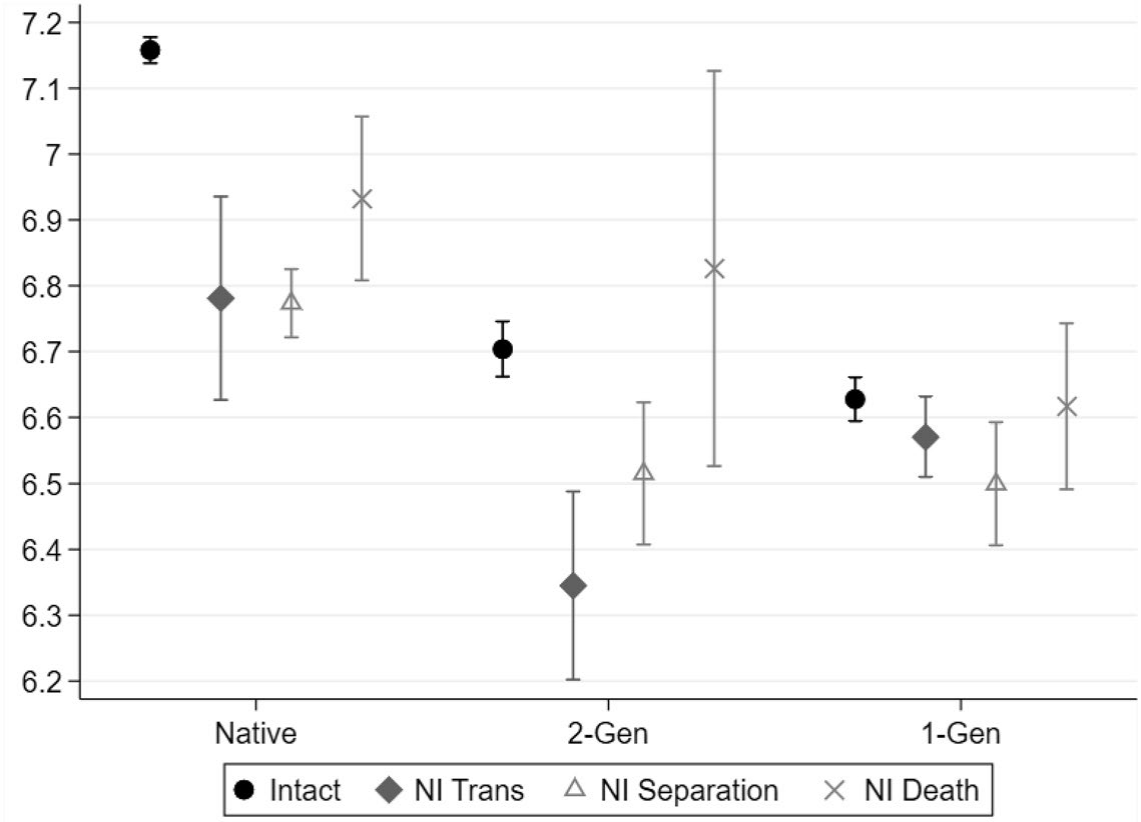
School report grades (mathematics), by family structure (detailed) and migration background (generation). *Note* Adjusted predictions after OLS model, with 83.5% C.I. Model controls for socio-demographic characteristics (Step 1). NI = non-intact. *N* = 31,046. Source: Integration of the Second Generation survey (ISTAT 2015)

Figure 3 analyses the non-intact penalty breaking down the variable according to the type of disruption experienced by the family: transnationalism (diamonds), separation (triangles), and death (crosses).

Natives living in different types of non-intact families consistently reported lower grades compared to their counterparts in intact families, with very limited differences based on the reason for parental absence. Among *2-Gen* children, the non-intact penalty was even stronger in cases of transnationalism compared to situations where the absent parent lived in Italy. This directly contradicts H4, which predicted a stronger non-intact penalty associated with separation than to transnationalism. *1-Gen* children achieved slightly higher grades when living in a transnational family, meaning that a statistically significant gap emerged between children in intact families and those in non-intact families due to parental separation. However, differences between family types were so small that they were of little significance.

Migrant children having experienced the death of one of their parents had virtually the same grades as those in intact families, and parental death was associated with a lower penalisation compared to other reasons for parental absence also among natives. Notwithstanding the high estimation uncertainty, this result aligns with previous evidence for Italy (Guetto & Panichella, 2019).

### Non-intact Family and Upper Secondary School Aspirations

**Fig. 4:**
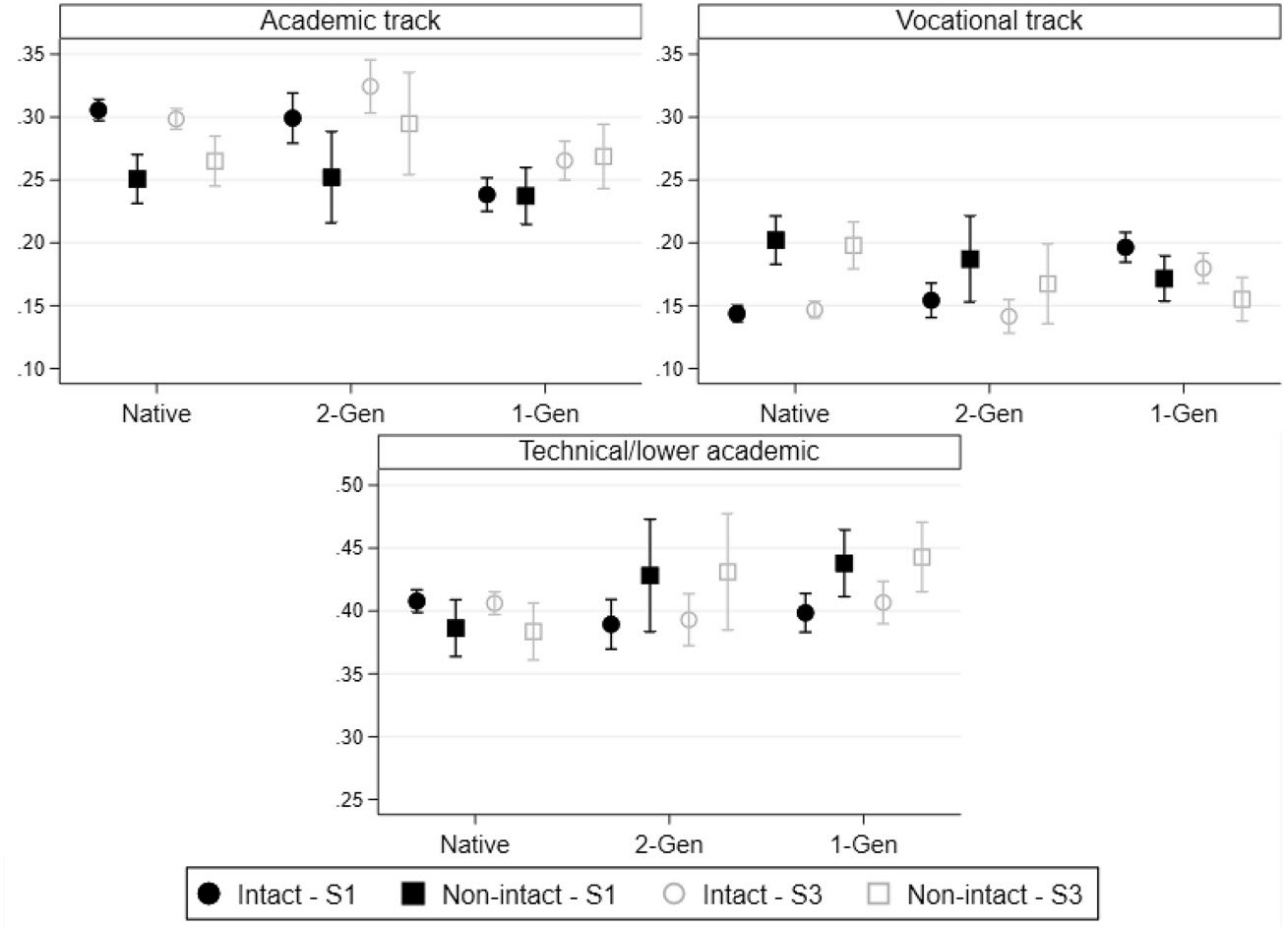
Upper secondary school aspirations, by family structure and migration background (generation). *Note* Predicted probabilities after multinomial logistic regression models, with 83.5% C.I Models control for socio-demographic characteristics (Step 1) and are augmented with the economic condition of the household (Step 2, not shown) and family environment (Step 3). *N* = 31,046. Source: Integration of the Second Generation survey (ISTAT 2015)

**Fig. 5:**
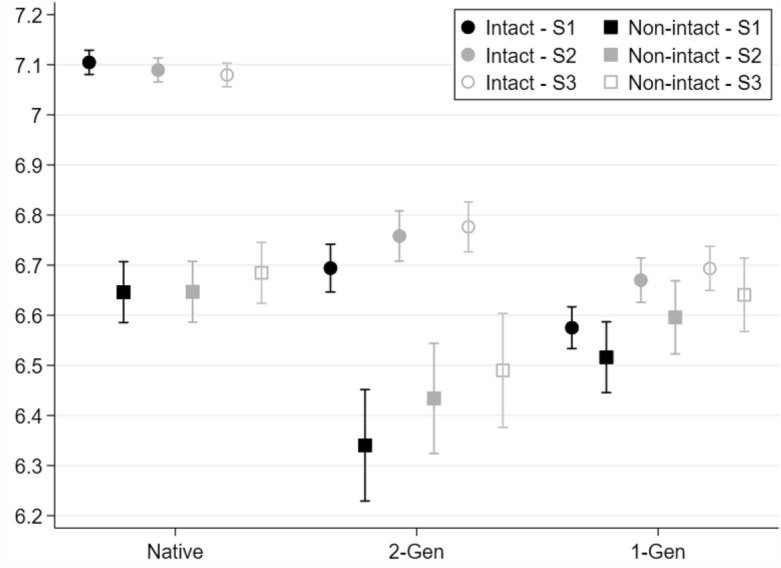
School report grades (Italian), by family structure and migration background (generation). *Note* Adjusted predictions after OLS models, with 83.5% C.I. Models control for socio-demographic characteristics (Step 1) and are augmented with the economic condition of the household (Step 2) and family environment (Step 3). *N* = 30,946. Source: Integration of the Second Generation survey (ISTAT 2015)

**Fig. 6:**
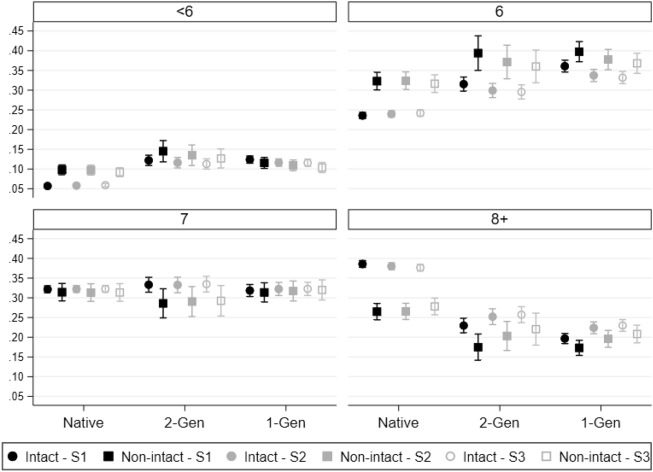
School report grades (mathematics) in categories, by family structure and migration background (generation). *Note* Predicted probabilities after multinomial logistic regression models, with 83.5% C.I. Models control for socio-demographic characteristics (Step 1) and are augmented with the economic condition of the household (Step 2) and family environment (Step 3). *N* = 31,046. Source: Integration of the Second Generation survey (ISTAT 2015)

The last step of the analysis concerned upper secondary school aspirations, namely in which track pupils sought to enrol following the end of lower secondary school. Figure 4 shows predicted probabilities after a multinomial logistic regression model, for the academic (upper left panel), the technical/lower academic (bottom panel), and the vocational track (upper right panel).^6^

Looking at the results of Step 1 (black symbols), which controlled for socio-demographic characteristics, including both parents’ education, native and *2-Gen* children had highly similar probabilities of expressing their intentions to enrol in the most prestigious academic tracks (approximately 30%), whereas *1-Gen* children still lagged behind (24%). However, upper secondary school aspirations were only significantly depressed among native children living in non-intact families, with a decline of slightly over 5 percentage points in academic track aspirations. *2-Gen* children also experienced a decline of approximately 4 percentage points in the likelihood of aspiring to the academic track, even if the confidence intervals overlapped. On the other hand, living in a non-intact family increased the likelihood of aspiring to the vocational track, by 6 percentage points, only among native children (upper right panel). We detected no statistically and substantially significant penalties as far as the technical/lower academic school track was concerned. These results thus confirm H1 and H2a when also considering a completely different educational outcome. It is worth underlining that, when living in a non-intact family, native children lose virtually all their advantage compared to *1-Gen* migrant children.

Similarly to the results concerning grades in mathematics, possible mediating variables related to household’s economic situation and family environment only explained the non-intact penalty found for native children to a very limited extent (white symbols). Interestingly, we found that, in Step 3, *1-Gen* migrants only had slightly lower aspirations to enrol in the academic track compared to natives, whereas *2-Gen* migrants’ aspirations were even higher than those of natives, regardless of their weaker performances at the time of the interview. This aligns with studies on how migrant families tend to hold unrealistically optimistic aspirations of their children’s educational trajectories (Salikutluk, 2016).

## Conclusions

In this paper, we have contributed to the literature on the consequences of non-intact families for children’s educational outcomes, with an empirical analysis focusing on children attending lower secondary school in Italy. In particular, we explored heterogeneity by migration background (previously underexplored in the literature), by comparing non-intact penalties for *Natives* (Italian-born children of at least one Italian-born parent), *2-Gen* migrant children (Italian-born children of two foreign-born parents), and *1-Gen* migrant children (foreign-born children of at least one foreign-born parent). Furthermore, we also took the children’s area of origin (Eastern Europe; East-South-and-Southeast Asia; Middle East and North Africa; Else) into consideration.

We formulated theoretical predictions based on a ‘more to lose’ mechanism, which has been previously proposed in relation to heterogeneity in the divorce penalty by parental SES, and applied to explain why the effects of parental separation are usually weaker among black/minority children, especially in North America. The argument is that high-SES and majority children are raised in households with more social, cultural, and economic resources than their low-SES and black/minority counterparts. As such, high-SES and majority children have more resources to lose because of parental separation, which is usually associated with deteriorating family environments and income losses. Conversely, the educational outcomes of low-SES and black/minority children are usually poorer to begin with, meaning that family disruptions can only marginally deteriorate them.

We argued that such a ‘more to lose’ mechanism may be usefully extended to the study of heterogeneity in non-intact penalties by migration background—especially in the Italian setting, characterised as it is by poor immigrants’ socioeconomic integration and strong native–migrant educational gaps. However, differences in the reasons behind non-intact families may also contribute to stronger non-intact penalties for native children than migrant ones. In fact, parental absence among migrant children is often related to transnationalism—which is part of a migratory project to improve the family’s prospects—whereas union dissolution is the most prevalent reason among natives, with its correlates of increased stress, family conflict, and economic difficulties.

Consistent with our expectations, we found natives to suffer negative consequences (in terms of both school grades and upper secondary school aspirations) from living in a single-parent household. Conversely, we found *1-Gen* migrant children to be unaffected by family structure, whereas *2-Gen* children were somewhat in between these two groups. These results are thus consistent with the ‘more to lose’ mechanism. In other words, native children lose part of their advantage (over their migrant peers) because of parental breakups. We found no substantial heterogeneity in the non-intact penalty by migrants’ areas of origin. This may be due to limited differences in the diffusion and acceptance of separation and single-parent households among migrant groups in Italy, and/or to the fact that different ethnic-specific mechanisms offset each other.

However, we were not able to provide robust evidence concerning the role of factors that were theoretically deemed to generate stronger non-intact penalties for native children. First, including in the models a rich set of variables concerning the economic condition of the household and the family environment only accounted for a minor share of the non-intact penalty and its heterogeneity by migration background. Second, transnationalism had a negative association with school grades, similar to parental separation among both native and *2-Gen* children. For *1-Gen* children, living in a single-parent household was almost inconsequential regardless of the non-intact family type.

These results could be due to different reasons. One could argue that parental economic resources are only relevant for more ambitious educational plans, such as university enrolment, rather than school performance at the lower secondary level. Additionally, more or less pronounced consequences of parental separation may have been induced by floor/ceiling effect in grades: only native pupils are likely to obtain excellent grades (e.g. 8 or more), meaning that there is more ‘room’ for them to be negatively affected by parental separation. On the contrary, lower secondary school teachers might be unwilling to assign failing grades in school reports, and especially to go below a certain threshold—indeed, only a tiny minority of children (fewer than 3%), including migrants, obtained a grade lower than 5.

Difficulties in accounting for the heterogeneity in non-intact penalties may have been due to methodological reasons, which is to say our study’s main limitations. First, we only indirectly operationalised the reasons for parental absence. While we feel confident in assuming parental separation/divorce for children whose absent parent lives in Italy but in a different house or city (especially among native children), the picture becomes more complicated for transnational families. In our data, transnationalism may also entail parental breakups, that is, parents may separate *and* one goes back to the origin country. This may account for the lack of differences in the effects of different types of non-intact family, particularly among *2-Gen* children, for whom we even detected a slightly stronger deterioration in school performance when living in a transnational family rather than other non-intact family types. Unfortunately, the data at hand did not allow us to account for this limitation. However, we were able to perform two robustness checks (the results of which are available upon request). First, we distinguished between *1-Gen* children who first came to Italy with both parents, and those who arrived with only one, to see whether they became a transnational family before or after migration; admittedly, the parent’s return to the country of origin after migration could have been due to a breakup. Second, we checked whether children living in a transnational household also cohabited with the co-resident parent’s new partner, which would benchmark separation from the absent parent. Both checks ensured the robustness of our results, as families that are potentially both separated *and* transnational did not substantially differ from transnational and other non-intact families.

A second limitation of this study is that parental education and other socioeconomic characteristics of the household were self-reported by the children. Considering their young age (on average, between 12 and 13), and that almost a fourth were non-native Italian speakers, this may have introduced a certain degree of measurement error, which reduced our variables’ explanatory power. Despite the questionnaire having been designed for children, they may have not been able to precisely assess the economic condition of their household, or the exact amount of resources available. Indeed, similar problems hold for the parents’ educational levels.

In conclusion, the present study has highlighted that living in a non-intact family is detrimental for children’s school performances in Italy—but mostly for native children. Parental separation does not represent an additional source of penalisation for already vulnerable social groups, such as immigrant children, in line with previous evidence for ethnic minorities (Kalmijn, 2010; McLanahan & Sandefur, 1994). On the contrary, it rather ‘equalises down’ in that it reduces the advantages of ethnic-majority children. Unfortunately, data limitations prevented us from fully uncovering the mechanisms underlying this heterogeneity in the non-intact penalty. Accordingly, additional efforts are required for implementing longitudinal surveys to support causal inferences, as well as the possibility to disentangle the economic, cultural, and social implications of parental separation leading to the worsening of children’s educational outcomes.

### Electronic supplementary material

Below is the link to the electronic supplementary material.

Supplementary file1 (PDF 343 kb)

## Data Availability

The data that support the findings of this study are available from the Italian National Institute of Statistics’ (ISTAT) website. A restricted version of the survey is publicly available for download from https://www.istat.it/it/archivio/210741. The complete data set can be obtained upon registration and appropriate request at the Contact Center https://contact.istat.it/.

## Appendix

**Table 4:** Descriptive statistics (control variables), by migration background (generation)

	Natives		2-Gen		1-Gen		Total	
	Mean; N	SD; *%*	Mean; N	SD; *%*	Mean; N	SD; *%*	Mean; N	SD; *%*
*Controls (1): socio-demographics*								
Female	8642	*49.3*	2669	*48.02*	3657	*45.95*	14,968	*48.21*
Age	12.34	1.00	12.37	1.06	13.11	1.19	12.54	1.11
Siblings	1.13	0.85	1.49	1.09	1.32	1.12	1.24	0.98
School year								
1st year (*prima media*)	5981	*34.12*	2141	*38.52*	2336	*29.35*	10,458	*33.69*
2nd year (*seconda media*)	5859	*33.42*	1847	*33.23*	2655	*33.36*	10,361	*33.37*
3rd year (*terza media*)	5689	*32.45*	1570	*28.25*	2968	*37.29*	10,227	*32.94*
Region								
North-West	3455	*19.71*	1395	*25.1*	1460	*18.34*	6310	*20.32*
North-East	5033	*28.71*	1738	*31.27*	2243	*28.18*	9014	*29.03*
Centre	3844	*21.93*	1412	*25.4*	1589	*19.96*	6845	*22.05*
South and Islands	5197	*29.65*	1013	*18.23*	2667	*33.51*	8877	*28.59*
Mother's education								
No title, elementary school	1204	*6.87*	998	*17.96*	1457	*18.31*	3659	*11.79*
Lower secondary school	3101	*17.69*	976	*17.56*	1355	*17.02*	5432	*17.5*
Upper secondary school	5209	*29.72*	1085	*19.52*	1770	*22.24*	8064	*25.97*
University	3542	*20.21*	561	*10.09*	840	*10.55*	4943	*15.92*
Don’t know	4473	*25.52*	1938	*34.87*	2537	*31.88*	8948	*28.82*
Father's education								
No title, elementary school	1422	*8.11*	1030	*18.53*	1619	*20.34*	4071	*13.11*
Lower secondary school	3724	*21.24*	849	*15.28*	1261	*15.84*	5834	*18.79*
Upper secondary school	4827	*27.54*	1095	*19.7*	1721	*21.62*	7643	*24.62*
University	2867	*16.36*	526	*9.46*	736	*9.25*	4129	*13.3*
Don’t know	4689	*26.75*	2058	*37.03*	2622	*32.94*	9369	*30.18*
*Controls (2): economic condition*								
Economic condition								
(Very) rich	4037	*23.03*	879	*15.82*	1293	*16.25*	6209	*20*
Neither rich, nor poor	12,945	*73.85*	4380	*78.81*	6164	*77.45*	23,489	*75.66*
(Very) poor	547	*3.12*	299	*5.38*	502	*6.31*	1348	*4.34*
Objects/appliances of the household	4.00	0.91	3.30	1.07	3.21	1.14	4	1.07
Housing condition								
Other kind of housing	684	*3.9*	392	*7.05*	624	*7.84*	1700	*5.48*
PPR < 0.76	11,216	*63.99*	1920	*34.54*	2578	*32.39*	15,714	*50.62*
PPR > = 0.76 & < = 1.24	4121	*23.51*	2004	*36.06*	2569	*32.28*	8694	*28*
PPR > 1.24	1508	*8.6*	1242	*22.35*	2188	*27.49*	4938	*15.91*
Count on someone (no)	861	*4.91*	590	*10.62*	1057	*13.28*	2508	*8.08*
*Controls (3): family environment*								
School involvement	4.29	0.77	4.02	0.85	4.04	0.81	4.18	0.71
Parenting quality	4.01	0.81	3.84	0.86	3.86	0.88	3.94	0.84
Punishment	3.63	0.82	3.60	0.84	3.65	0.86	3.63	0.83
Indifference	4.50	0.75	4.34	0.85	4.23	0.92	4.40	0.82
Total	17,529	*100*	5,558	*100*	7,959	*100*	31,046	*100*

Mean and standard deviation (SD) are reported for continuous variables; cell size (N) and column percentages (*%*, in italics) are reported for categorical variables. *N* = 31,046. Unweighted data. Source: Integration of the Second Generation survey (ISTAT 2015)

## Footnotes

1 Italian schools usually provide mid-year school reports between January and February, whereas the children were interviewed between March and June. As we had no information on the timing of changes in family arrangements, these may have occurred *after* children received the last school report, which is based on teachers’ evaluations during the first term. However, given the short time period, the number of cases involved should be very limited. The analysis of upper secondary school aspirations, our second educational outcome, was unaffected by this timing issue.

2 Unfortunately, we had no information on the timing of changes in family arrangements.

3 We tried to distinguish between children who arrived in Italy before reaching the age of 10 (*N* = 6468) and those who arrived after (*N* = 1491); that is, between those who attended elementary school in Italy and those who did not. The two groups closely resembled each other in grades and non-intact penalties.

4 Figure 1 reports 83.5% confidence intervals around predictions, so that overlapping intervals indicate a lack of statistical significance at the 5% level (Cumming 2009).

5 Children in single-parent households tend to live with their mothers in most cases. However, we found no differences in school grades between children living only with the mother or the father (results available upon request).

6 For ease of interpretation, we do not show results for the ‘don’t know’ and ‘else’ response categories, as non-intact penalties were virtually null and differences across models very small. However, the full results can be found in Table S4 in supplementary materials.
