# Supplementary material for: Non-intact Families and Children’s Educational Outcomes: Comparing Native and Migrant Pupils
Source: Eur J Popul. 2022 Sep 5;38(5):1065–94. doi: 10.1007/s10680-022-09638-z (PMC9727002; doi:10.1007/s10680-022-09638-z)
Supplement: Supplementary file 1 — Supplementary file1 (PDF 343 kb) [file 10680_2022_9638_MOESM1_ESM.pdf]

Non-intact families and children's educational outcomes: comparing native and migrant pupils

Supplementary material

Table S1 Linear regression models for the relation between school report grades (mathematics), family structure, and migration background (generation), stepwise approach

	Step 1			Step 2			Step 3		
	Coef.		SE	Coef.		SE	Coef.		SE
Migration background: ref. Native									
2-Gen	-0.46	***	0.03	-0.37	***	0.04	-0.35	***	0.04
1-Gen	-0.53	***	0.03	-0.43	***	0.03	-0.40	***	0.03
Non-intact family	-0.35	***	0.04	-0.33	***	0.04	-0.28	***	0.04
2-Gen # Non-intact family	0.15	*	0.07	0.16	*	0.07	0.14	*	0.07
1-Gen # Non-intact family	0.28	***	0.05	0.26	***	0.05	0.24	***	0.05
Controls 1): Socio-demographics									
Female	0.37	***	0.02	0.36	***	0.02	0.33	***	0.02
Age	-0.09	***	0.02	-0.09	***	0.02	-0.07	***	0.02
Siblings	-0.07	***	0.01	-0.05	***	0.01	-0.02		0.01
School grade: ref. 1st year (*prima media*)									
2nd year (*seconda media*)	-0.02		0.03	-0.03		0.03	-0.04		0.03
3rd year (*terza media*)	0.05		0.05	0.03		0.05	0.03		0.05
Region: ref. North-West									
North-East	-0.06	*	0.03	-0.08	*	0.03	-0.08	*	0.03
Center	-0.22	***	0.03	-0.22	***	0.03	-0.23	***	0.03
South & Islands	-0.35	***	0.03	-0.34	***	0.03	-0.37	***	0.03
Mothers' Education: ref. NT									
LS	0.09		0.10	0.11		0.09	0.06		0.09
US	0.27	*	0.12	0.22		0.12	0.20		0.12
U	0.45	**	0.14	0.39	**	0.14	0.34	*	0.13
DK	-0.03		0.08	-0.05		0.08	-0.06		0.08
Fathers' Education: ref. NT									
LS	-0.14		0.09	-0.14		0.09	-0.14		0.10
US	0.36	**	0.11	0.31	**	0.11	0.24	*	0.11
U	0.62	***	0.17	0.58	***	0.16	0.54	**	0.16
DK	-0.04		0.10	-0.06		0.10	-0.05		0.10

(continued)

Table S1 (continued) Linear regression models for the relation between school report grades (mathematics), family structure, and migration background (generation), stepwise approach

	Step 1			Step 2			Step 3		
	Coef.		SE	Coef.		SE	Coef.		SE
Interaction term: Mother's # Father's Education									
LS # LS	0.18		0.13	0.13		0.13	0.12		0.13
LS # US	-0.06		0.15	-0.10		0.15	-0.04		0.14
LS # U	-0.35		0.22	-0.39		0.22	-0.36		0.21
LS # DK	0.10		0.15	0.07		0.15	0.04		0.15
US # LS	0.40	*	0.15	0.39	*	0.15	0.32	*	0.15
US # US	0.11		0.16	0.12		0.16	0.10		0.16
US # U	0.00		0.21	-0.01		0.21	-0.05		0.21
US # DK	0.17		0.17	0.20		0.18	0.11		0.17
U # LS	0.19		0.18	0.18		0.18	0.15		0.17
U # US	0.02		0.18	0.03		0.18	0.04		0.17
U # U	-0.12		0.22	-0.12		0.22	-0.14		0.21
U # DK	0.23		0.18	0.24		0.18	0.19		0.18
DK # LS	0.37	**	0.14	0.36	**	0.14	0.31	*	0.14
DK # US	0.00		0.15	0.01		0.14	0.03		0.14
DK # U	-0.04		0.20	-0.06		0.19	-0.08		0.19
DK # DK	0.29	*	0.12	0.29	*	0.12	0.23		0.12
Controls 2): Economic condition									
Economic condition: ref. (very) rich									
Neither rich, nor poor				-0.07	*	0.03	-0.07	*	0.03
(very) poor				-0.17	**	0.06	-0.11		0.06
Objects/appliances of the household				0.04	**	0.01	0.03	**	0.01
Housing: ref. PPR>=0.76 & PPR<=1.24									
Other kind of housing				-0.05		0.06	0.00		0.06
PPR<0.76				0.14	***	0.03	0.13	***	0.03
PPR>1.24				-0.13	**	0.04	-0.10	**	0.04
Count on someone (no)				-0.17	***	0.04	-0.09	*	0.04
Controls 3): Family environment									
School Involvement							0.06	***	0.02
Parenting quality							0.16	***	0.02
Punishment							0.05	**	0.01
Indifference							0.12	***	0.02
Constant	7.90	***	0.23	7.70	***	0.23	5.95	***	0.26

Source: Integration of the Second Generation survey (ISTAT 2015). N=31,046.

Note: Full models referring to Figure 1. Ref = reference. NT= No title, elementary school; LS= Lower secondary school; US= Upper secondary school; U= University; DK= Don't know. *p< .05, **p< .01, ***p< .001

Table S2 Linear regression models for the relation between school report grades (mathematics), family structure, and migration background (area of origin & generation)

	Coef.		SE
Migration background: ref. Native			
2-Gen: EastEU	-0.34	***	0.05
2-Gen: S/E/SEAsia	-0.22	***	0.05
2-Gen: MENA	-0.44	***	0.08
2-Gen: Else	-0.42	***	0.07
1-Gen: EastEU	-0.39	***	0.03
1-Gen: S/E/SEAsia	-0.36	***	0.06
1-Gen: MENA	-0.38	***	0.06
1-Gen: Else	-0.49	***	0.06
Non-intact family	-0.28	***	0.04
2-Gen: EastEU # Non-intact family	0.19		0.11
2-Gen: S/E/SEAsia # Non-intact family	0.14		0.16
2-Gen: MENA # Non-intact family	0.27		0.16
2-Gen: Else # Non-intact family	0.13		0.12
1-Gen: EastEU # Non-intact family	0.24	***	0.06
1-Gen: S/E/SEAsia # Non-intact family	0.34	**	0.10
1-Gen: MENA # Non-intact family	0.28	*	0.13
1-Gen: Else # Non-intact family	0.26	**	0.09
Controls 1): Socio-demographics			
Female	0.33	***	0.02
Age	-0.07	***	0.02
Siblings	-0.02		0.01
School grade: ref. 1st year (*prima media*)			
2nd year (*seconda media*)	-0.04		0.03
3rd year (*terza media*)	0.03		0.05
Region: ref. North-West			
North-East	-0.08	*	0.03
Center	-0.23	***	0.03
South & Islands	-0.37	***	0.03
Mothers' Education: ref. NT			
LS	0.06		0.09
US	0.20		0.12
U	0.34	*	0.13
DK	-0.06		0.08
Fathers' Education: ref. NT			
LS	-0.14		0.10
US	0.25	*	0.11
U	0.55	***	0.16
DK	-0.04		0.10

(continued)

Table S2 (continued) Linear regression models for the relation between school report grades (mathematics), family structure, and migration background (area of origin & generation)

	Coef.		SE
Interaction term: Mother's # Father's Education			
LS # LS	0.12		0.13
LS # US	-0.05		0.14
LS # U	-0.37		0.21
LS # DK	0.04		0.15
US # LS	0.32	*	0.15
US # US	0.09		0.16
US # U	-0.06		0.21
US # DK	0.10		0.17
U # LS	0.15		0.17
U # US	0.04		0.17
U # U	-0.15		0.21
U # DK	0.19		0.18
DK # LS	0.31	*	0.14
DK # US	0.02		0.14
DK # U	-0.09		0.19
DK # DK	0.22		0.12
Controls 2): Economic condition			
Economic condition: ref. (very) rich			
Neither rich, nor poor	-0.07	*	0.03
(very) poor	-0.11		0.06
Objects/appliances of the household	0.03	**	0.01
Housing: ref. PPR>=0.76 & PPR<=1.24	0.00		.
Other kind of housing	0.00		0.06
PPR<0.76	0.13	***	0.03
PPR>1.24	-0.10	**	0.04
Count on someone (no)	-0.09	*	0.04
Controls 3): Family environment			
School Involvement	0.07	***	0.02
Parenting quality	0.16	***	0.02
Punishment	0.05	**	0.01
Indifference	0.12	***	0.02
Constant	5.95	***	0.26

Source: Integration of the Second Generation survey (ISTAT 2015). N=31,046.

Note: All control variables included (Step 3). Full models referring to Figure 2. Ref = reference. NT= No title, elementary school; LS= Lower secondary school; US= Upper secondary school; U= University; DK= Don't know. *p< .05, **p< .01, ***p< .001

Table S3 Linear regression models for the relation between school report grades (mathematics), family structure (detailed), and migration background (generation)

	Coef.		SE
Migration background: ref. Native			
2-Gen	-0.45	***	0.03
1-Gen	-0.53	***	0.03
Types of non-intact family: ref. intact			
Non-intact: Transnational	-0.38	***	0.11
Non-intact: Separation	-0.39	***	0.04
Non-intact: Death	-0.23	*	0.09
Non-intact: Don't know	-0.04		0.19
2-Gen # Non-intact: Transnational	0.02		0.15
2-Gen # Non-intact: Separation	0.20	*	0.09
2-Gen # Non-intact: Death	0.35		0.24
2-Gen # Non-intact: Don't know	-0.04		0.23
1-Gen # Non-intact: Transnational	0.32	**	0.12
1-Gen # Non-intact: Separation	0.26	**	0.08
1-Gen # Non-intact: Death	0.22		0.13
1-Gen # Non-intact: Don't know	0.03		0.21
Controls 1): Socio-demographics			
Female	0.37	***	0.02
Age	-0.09	***	0.02
Siblings	-0.07	***	0.01
School grade: ref. 1st year (*prima media*)			
2nd year (*seconda media*)	-0.02		0.03
3rd year (*terza media*)	0.05		0.05
Region: ref. North-West			
North-East	-0.06		0.03
Center	-0.22	***	0.03
South & Islands	-0.35	***	0.03
Mothers' Education: ref. NT			
LS	0.09		0.10
US	0.26	*	0.12
U	0.44	**	0.14
DK	-0.04		0.08
Fathers' Education: ref. NT			
LS	-0.13		0.09
US	0.36	**	0.11
U	0.62	***	0.17
DK	-0.04		0.10

(continued)

Table S3 (continued) Linear regression models for the relation between school report grades (mathematics), family structure (detailed), and migration background (generation)

	Coef.		SE
Interaction term: Mother's # Father's Education			
LS # LS	0.19		0.13
LS # US	-0.06		0.15
LS # U	-0.33		0.22
LS # DK	0.10		0.15
US # LS	0.41	**	0.15
US # US	0.12		0.16
US # U	0.02		0.21
US # DK	0.19		0.17
U # LS	0.20		0.18
U # US	0.03		0.18
U # U	-0.10		0.22
U # DK	0.26		0.18
DK # LS	0.38	**	0.14
DK # US	0.01		0.15
DK # U	-0.03		0.20
DK # DK	0.31	*	0.12
Constant	7.90	***	0.22

Source: Integration of the Second Generation survey (ISTAT 2015). N=31,046.

Note: Socio-demographic control variables included (Step 1). Full models referring to Figure 3. Ref = reference. NT= No title, elementary school; LS= Lower secondary school; US= Upper secondary school; U= University; DK= Don't know. *p< .05, **p< .01, ***p< .001

Table S4 Multinomial logistic regression models for the relation between upper secondary school aspirations, family structure, and migration background (generation). Base outcome: academic track

	Technical/Lower academic			Vocational Track			Else			Don't know		
	Coef.		SE	Coef.		SE	Coef.		SE	Coef.		SE
	base outcome: academic track											
Migration background: ref. Native												
2-Gen	-0.13		0.09	-0.14		0.12	-0.09		0.13	-0.24		0.14
1-Gen	0.14		0.08	0.36	***	0.10	0.23		0.12	0.00		0.13
Non-intact family	0.08		0.10	0.48	***	0.12	0.05		0.15	0.35	*	0.16
2-Gen # Non-intact family	0.12		0.21	-0.18		0.26	-0.34		0.28	-0.42		0.30
1-Gen # Non-intact family	-0.02		0.15	-0.66	***	0.18	-0.11		0.22	-0.65	**	0.24
Controls 1): Socio-demographics												
Female	0.46	***	0.06	0.09		0.07	0.24	**	0.09	-0.11		0.10
Age	0.05		0.05	0.29	***	0.06	0.32	***	0.07	0.56	***	0.09
Siblings	0.03		0.03	0.12	**	0.04	0.16	**	0.05	0.01		0.05
School year: ref. 1st year (*prima media*)												
2nd year (*seconda media*)	0.13		0.08	0.25	*	0.11	-0.19		0.13	-0.81	***	0.15
3rd year (*terza media*)	0.40	***	0.12	0.33	*	0.15	-1.02	***	0.19	-2.56	***	0.26
Region: ref. North-West												
North-East	0.36	***	0.08	0.19		0.10	0.12		0.12	-0.28	*	0.13
Center	-0.03		0.08	-0.23	*	0.11	-0.33	*	0.13	-0.22		0.14
South & Islands	-0.24	**	0.08	-0.28	**	0.10	-0.45	***	0.12	-0.05		0.13
Mothers' Education: ref. NT												
LS	0.17		0.31	0.45		0.30	-0.15		0.38	0.28		0.40
US	-0.31		0.34	-0.58		0.37	-0.22		0.45	-0.88		0.46
U	-0.25		0.38	-0.77		0.43	-0.53		0.49	-0.46		0.42
DK	-0.10		0.27	0.23		0.31	0.00		0.34	-0.02		0.30
Fathers' Education: ref. NT												
LS	0.01		0.34	0.18		0.36	-0.06		0.44	0.34		0.38
US	0.19		0.36	-0.03		0.38	-0.69		0.47	-0.26		0.42
U	-0.18		0.44	-0.84		0.47	0.33		0.56	0.28		0.55
DK	0.04		0.29	-0.18		0.29	0.15		0.35	0.18		0.42

(continued)

Table S4 (continued) Multinomial logistic regression models for the relation between upper secondary school aspirations, family structure, and migration background (generation). Base outcome: academic track

	Technical/Lower academic			Vocational Track			Else			Don't know		
	Coef.		SE	Coef.		SE	Coef.		SE	Coef.		SE
	base outcome: academic track											
Interaction term: Mother's # Father's Education												
LS # LS	0.32		0.43	-0.15		0.45	0.38		0.57	-0.53		0.54
LS # US	0.10		0.46	-0.25		0.48	0.65		0.61	-0.51		0.61
LS # U	-0.17		0.57	-0.61		0.64	-1.68	*	0.76	-1.14		0.76
LS # DK	0.08		0.46	-0.40		0.47	0.09		0.56	-0.42		0.64
US # LS	0.43		0.46	0.03		0.50	-0.29		0.63	-0.64		0.60
US # US	0.07		0.47	-0.49		0.51	0.18		0.63	-0.23		0.62
US # U	-0.11		0.54	-0.57		0.61	-1.50	*	0.73	-1.27		0.84
US # DK	0.25		0.45	0.45		0.48	-0.23		0.60	-0.19		0.67
U # LS	0.14		0.52	0.32		0.59	0.56		0.70	-0.30		0.64
U # US	-0.43		0.50	-0.75		0.59	0.12		0.67	-1.33		0.69
U # U	-0.50		0.56	-0.56		0.64	-0.89		0.73	-1.33		0.70
U # DK	-0.11		0.48	-0.34		0.54	-0.24		0.60	-0.79		0.65
DK # LS	0.51		0.47	-0.29		0.52	0.04		0.70	-0.67		0.55
DK # US	-0.25		0.45	-0.48		0.52	-0.08		0.65	-0.72		0.61
DK # U	-0.03		0.54	-1.04		0.64	-1.19		0.73	-1.16		0.69
DK # DK	-0.02		0.36	-0.40		0.39	-0.18		0.44	-0.70		0.50
Controls 2): Economic condition												
Economic condition: ref. (very) rich												
Neither rich, nor poor	0.26	***	0.07	0.29	**	0.10	0.40	***	0.11	0.16		0.12
(very) poor	0.14		0.17	0.12		0.20	0.53	*	0.26	0.35		0.22
Objects/appliances of the household	-0.06		0.03	-0.11	**	0.04	-0.05		0.05	-0.18	***	0.05
Count on someone (no)	-0.06		0.13	0.05		0.15	0.14		0.19	0.59	**	0.18
Housing: ref. PPR>=0.76 & PPR<=1.24												
Other kind of housing	0.12		0.16	0.16		0.19	0.22		0.22	0.70	***	0.21
PPR<0.76	0.01		0.07	-0.23	*	0.09	0.19		0.11	-0.21		0.12
PPR>1.24	0.05		0.11	0.06		0.13	0.34	*	0.15	0.32	*	0.15
Controls 3): Family environment												
School Involvement	-0.09	*	0.04	-0.18	***	0.05	-0.13	*	0.06	-0.44	***	0.06
Parenting quality	-0.22	***	0.04	-0.30	***	0.05	-0.27	***	0.06	-0.44	***	0.07
Punishment	0.01		0.04	0.00		0.05	0.08		0.06	-0.06		0.06
Indifference	-0.21	***	0.05	-0.34	***	0.06	-0.33	***	0.07	-0.49	***	0.07
Constant	1.58	*	0.69	-0.22		0.82	-2.15	*	0.99	-0.19		1.23

Source: Integration of the Second Generation survey (ISTAT 2015). N=31,046.

Note: All control variables included (Step 3). Full models referring to Figure 4. Ref = reference. NT= No title, elementary school; LS= Lower secondary school; US= Upper secondary school; U= University; DK= Don't know. *p< .05, **p< .01, ***p< .001
